# Intraocular pressure measurement using ICare rebound tonometer in different positions of eye and different locations on cornea

**DOI:** 10.1097/MD.0000000000034874

**Published:** 2023-09-08

**Authors:** Sirada Wongwanwatana, Isaraporn Treesit, Panrapee Funarunart, Wallop Iemsomboon, Raveewan Choontanom

**Affiliations:** a Department of Ophthalmology, Phramongkutklao Hospital, Phramongkutklao College of Medicine, Bangkok, Thailand.

**Keywords:** IC200, iCare rebound tonometer, intraocular pressure

## Abstract

Intraocular pressure (IOP) is one of the most crucial aspects for diagnosis and treatment plan among patients with glaucoma. Although the gold standard for IOP measurement is Goldmann applanation tonometer (GAT)^[1]^, it must be mounted to a slit lamp biomicroscope. However, rebound tonometer has become popular due to its ease of operation and portable design, does not require topical anesthesia, and results do not differ significantly from those of GAT^[2]^.

The purpose of this cross-sectional study is to investigate the difference in IOP measurement with iCare IC200 in different angles of the eye and different corneal locations. All participants underwent IOP measurement by GAT twice. Then, IOP was measured with iCare by a single physician. IOP was measured in a straight manner in the upright patient position; then participants were asked to look at fixation targets, which located in four different points. IOP was measured in upgaze, downgaze, medial gaze, and lateral gaze. Then, IOP was measured at 2 mm from limbus in superior, inferior, nasal, and temporal cornea. All methods were measured twice, and the mean was used for calculation. The physician who measured IOP by iCare was masked from GAT results. A total of 168 eyes were tested with a mean age of 62.15 ± 12.34 years. Mean IOP measured by GAT and iCare at the central cornea was 15.53 ± 5.57 and 14.78 ± 6.14 mmHg, respectively. The standardized mean difference (SMD) between iCare and GAT was 0.13 (-0.09-0.34), which is insignificant. The average IOP was 0.6, 0.47, 0.91, and 0.44 mmHg lower than the primary position in upgaze, downgaze, medial gaze, and lateral gaze 15 degrees angulated positions respectively (*p*<.01). IOPs at 2 mm from limbus in the inferior, nasal, and temporal cornea were 0.5, 0.69, and 0.57 mmHg lower than IOP measured at the central cornea (*p*=<.01). IOP measurements with iCare in different angles of eye were statistically significantly lower than in the primary position. Similarly, IOPs at different locations on cornea were lower than at the central cornea. However, the difference in IOP measurements with iCare in different angles of the eye and different corneal locations was in the trivial range and might be clinically insignificant.

## 1. Introduction

Intraocular pressure (IOP) is one of the most crucial aspects for diagnosis and treatment plan among patients with glaucoma. Although the gold standard for IOP measurement is Goldmann applanation tonometer (GAT),^[1]^ it must be mounted to a slit lamp biomicroscope. However, rebound tonometer has become popular worldwide due to its ease of operation and portable design. In addition, rebound tonometer does not require topical anesthesia and fluorescein stain and the results do not differ significantly from GAT^[2]^.

The iCare IC200 is a rebound tonometer recently developed in 2020, weighing around 260 gm, probe size 1.8 mm and can measured IOP between 7 and 50 mm Hg with good reliability. To make a measurement, the probe needs to be held perpendicular to the surface of the cornea and the distance between the tip of the probe and the eye is about 4 to 8 millimeters.^[3]^ When the alignment of the tonometer and the probe are correct, the probe base indicator will emit a green light to allow measurement. Once 6 measurements have been successfully made, the tonometer will emit 1 long beep sound then the final IOP is displayed on the display screen in mm Hg. The values from the first to the fifth measurements are displayed. The 6th value is the running average value and constitutes the final IOP. The mean IOP difference between iCare IC200 and GAT was 1.27 mm Hg with a tendency to overestimate IOP when measured with iCare IC200 with 95% limits of agreement (LoA) ranging from −3.4 to 0.9 mm Hg for all ranges of IOP.^[4]^ The agreement between the IOP measurement by GAT and iCare IC200 was < 2 mm Hg at all ranges of IOP.^[4]^ Repeatability (coefficient of variation) of iCare IC200 was < 8%^[5]^.

One of the advantages of the rebound tonometer is that the area of applanation on the cornea is small, so this could be used when measurements cannot be taken from the central cornea by GAT. Furthermore, it can measure IOP in positions other than upright. Many ophthalmologists prefer to use a rebound tonometer among patients with special conditions such as a small palpebral fissure, an uncooperative client or those with a disability. However, its small probe diameter and variety of measurement conditions lead to both intentional and accidental measurement errors because the IOP is not measured at the central cornea in the primary eye position. IOP measurement in such cases may affect the results. Currently no study has focused on this issue.

This study investigated the difference in IOP values in different positions of the eye and different locations on the cornea. This study also examined the correlation between IOP and corneal thickness in that region, which was measured by a Zeiss Cirrus 5000 HD-OCT with an anterior segment lens.

## 2. Materials and methods

### 2.1. Study participants

This constitutes a cross-sectional study conducted at the Ophthalmic Outpatient Department, Phramongkutklao Hospital, Bangkok, Thailand, between May 2021 and August 2022. The study was approved by the Institutional Review Board of the Royal Thai Army Medical Department and adhered to the tenets of the Declaration of Helsinki. This trial was registered in the Thai Clinical Trial Registry and its identification number is TCTR20220207003. Informed consent was obtained from all participants before the study.

Inclusion criteria included participants with age older than 20. Exclusion criteria comprised subjects having a history of corneal disease that precluded measurement of IOP by GAT, the presence of any corneal scarring or tight orbit that could affect IOP measurement, a history of ocular trauma, a history of refractive surgery, thyroid eye disease, CN 3, 4, 6 palsy or limited version, strabismus, allergy to 0.5% tetracaine hydrochloride or fluorescein dye, or pregnancy.

### 2.2. IOP and central corneal thickness (CCT) measurement

**Figure 1.**
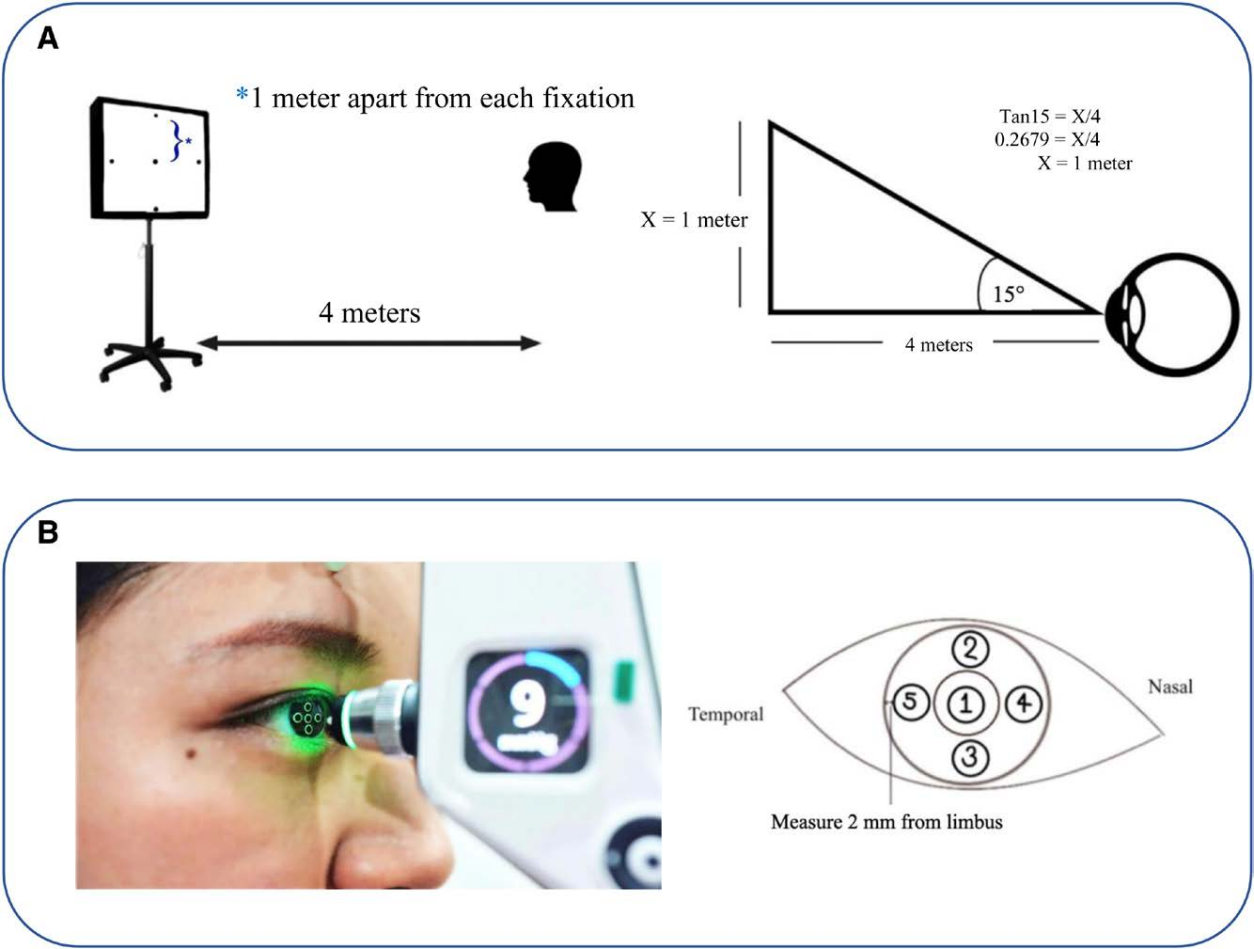
Fixation target models for IOP measurement using the iCare IC200 at 15-degree deviated angles (A) and pictures demonstrating different locations on the cornea measured by iCare IC200 (B). IOP = intraocular pressure.

All participants underwent IOP measurement by GAT twice. When IOP measurements had more than 3 mm Hg of difference, a third measurement was obtained. The average IOP was used as the final result. Then IOP was measured using the iCare IC200 (Icare Finland Oy, Helsinki, Finland) by a single physician under the following conditions: The first step was to measure IOP in an upright patient position along a straight line at the fixed eye axis. Participants were sitting 4 meters away from the fixation point. In the second step, participants were asked to look at fixation targets, which were located at 4 different points. This caused the eyes to shift 15 degrees from their primary position. Third, IOP was measured at different spots on the cornea, namely, 2 millimeters from the limbus in the superior, inferior, nasal, and temporal regions (Fig. 1). All methods were measured twice, and the mean was used for calculation. The physician measuring IOP by iCare was masked from the GAT results. The time between GAT and iCare measurements exceeded 30 minutes.

**Figure 2.**
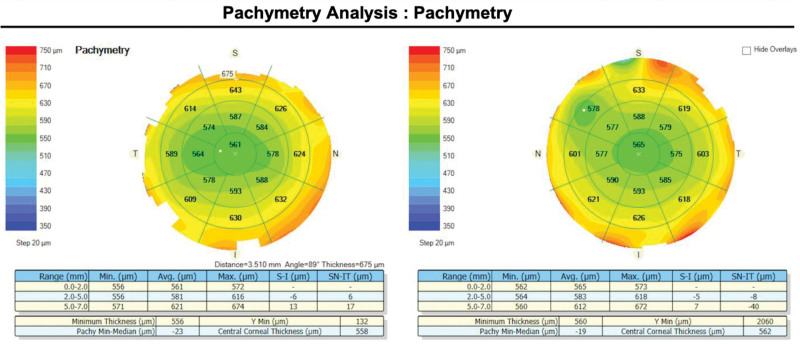
Pachymetry analysis shows the cornea thickness. For example, in the right eye, we used corneal thickness at the superior 7 mm point (OD = 675 µm) to analyze with IOP measured using the iCare IC200 at 2 mm from the superior limbus. IOP = intraocular pressure.

The corneal thickness readings were obtained from participants using the Zeiss Cirrus 5000 HD-OCT (Carl Zeiss Meditec, Dublin, CA) with a cornea lens for a 9 mm diameter pachymetry map. The corneal thickness of 7 mm, using the average corneal diameter of approximately 11 to 12 mm horizontally and 10 to 11 mm vertically, was selected for subsequent corneal thickness analysis and correlated to the peripheral cornea measured using the iCare IC200 at 2 millimeters from the limbus in the superior, inferior, nasal and temporal regions (Fig. 2). We assumed that corneal thickness at 7 mm diameter at 12, 6, 3, and 9 o’clock was in the same area as positions at 2 mm from the limbus in the superior, inferior, nasal and temporal cornea regions where the IOP was measured.

### 2.3. Statistical analysis

A minimum of 167 eyes were required for the calculation with a power of 0.8. Demographic data were analyzed by mean and standard deviation (SD) for continuous data and frequency (%) for discrete data. A paired *t* test was used to compare the average measurement of IOP using the iCare IC200 in different positions of the eye and different locations on the cornea. The receiver operating characteristic (ROC) curve and area under the ROC curve (AUC) were used to analyze all the possible decision thresholds from iCare IC200. The AUC is an overall summary of diagnostic accuracy. AUC equals 1 when it had perfect accuracy. Likelihood ratio (LR) was calculated from ROC curve. If the cut point had LR > 10 or < 0.1, it would be excellent diagnostic value. The agreement between IOP measured by the iCare IC200, the GAT and the Bland-Altman plot were used to analyze the data based on a 95% LoA. The LoA was calculated using an average difference of 1.96 SD. When the differences were < 1.96 SD, a good correlation was noted between the 2 methods. The correlation between CCT and IOP was investigated using linear regression. A *P* value of.05 was considered statistically significant, and *P* = .001 was considered statistically highly significant. The intraclass correlation coefficient (ICC) was applied for intra-observer reliability between both the iCare IC200 and GAT separately. In general, an ICC value > .8 indicates good repeatability, and a value > .9 suggests excellent repeatability of measurements.

All statistical analyses were performed using STATA Software, Version 17.0 (Stata Corp, College Station, TX) and MedCalc Software, Version 16.8.4.

## 3. Results

**Figure 3.**
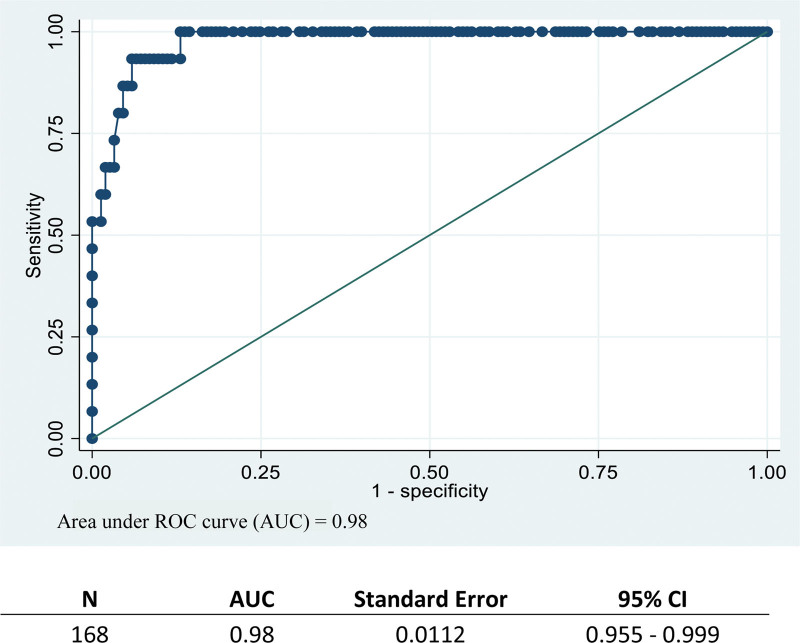
The Receiver Operating Characteristic (ROC) curve and area under the curve (AUC) values.

**Figure 4.**
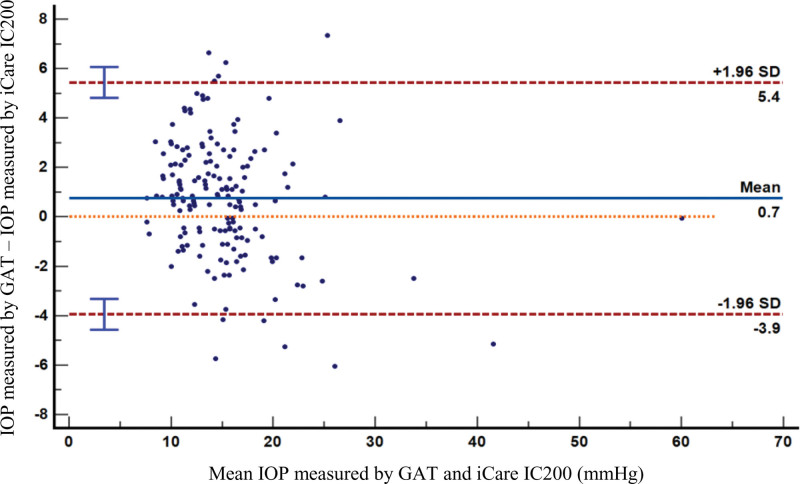
Bland-Altman plots of IOP measurement differences between iCare IC200 and GAT against the average of 2 measurements. Mean IOP difference was 0.7 mm Hg. GAT = Goldmann applanation tonometer, IOP = intraocular pressure.

A total of 168 eyes from 84 participants with a mean age of 62.15 ± 12.34 years old were recruited; both eyes of each individual were included. Seventy-eight eyes (46.43%) were male. Of the 168 eyes examined, 74 (44.05%) were healthy, 9 (5.4%) had ocular hypertension and 85 (50.6%) had glaucoma (Table 1). The mean IOP measured by GAT was 15.53 ± 5.57 mm Hg (14.68–16.38) and mean IOP measured by the iCare IC200 at the central cornea was 14.78 ± 6.14 mm Hg (13.85–15.72) (Table 2). The standardized mean difference of this difference was 0.13 (−0.09 to 0.34) which is insignificant. The minimal detectable change of iCare IC200 was 3.38 (2.88–3.93) and standard error of measurement was 1.22. The ROC curve showed accuracy of iCare IC200 was 94.05% and AUC was 0.98 (0.95–0.99). The most appropriate cutoff for differentiating by this curve for iCare IC200 is at 18.6 mm Hg which positive LR+, negative LR−, sensitivity and specificity were 15.87, 0.07, 93.33%, and 94.12% consecutively (Fig. 3). Bland-Altman plots correlated well between the 2 measurement methods (Fig. 4). The mean CCT was 520.60 ± 35.08 µm (Table 3).

**Table 1:** Demographics data.

Total = 168 eyes	N (%)
Sex	
Male	78 (46.40)
Female	90 (53.60)
Age (yr)	
Mean ± SD	62.15 ± 12.35
Diagnosis	
Normal	74 (44.05)
OHT	9 (5.40)
Glaucoma	85 (50.60)

SD = standard deviation.

**Table 2:** Average intraocular pressure by iCare IC200 in different positions of eye and different locations on cornea.

	Mean ± SD	*P* value
Icare ic200 at center	14.78 ± 6.14	
GAT	15.530 ± 5.57	<.001*
iCare IC200 upgaze 15°	14.18 ± 6.43	<.001*
iCare IC200 downgaze 15°	14.31 ± 6.28	<.001*
iCare IC200 medial gaze 15°	13.87 ± 6.25	<.001*
iCare IC200 lateral gaze 15°	14.34 ± 6.17	<.001*
iCare IC200 2mm from limbus at superior	14.75 ± 6.30	.825
iCare IC200 2mm from limbus at inferior	14.28 ± 6.06	<.001*
iCare IC200 2mm from limbus at nasal	14.09 ± 6.30	<.001*
iCare IC200 2mm from limbus at temporal	14.21 ± 6.48	<.001*

GAT = Goldmann applanation tonometer, SD = standard deviation.

* Paired *t* tests analysis.

**Table 3:** Corneal thickness in different positions on cornea.

Corneal thickness (µm)	Mean ± SD	95% CI	Median	Minimum	Maximum
Center	520.60 ± 35.08	515.25–525.94	520.50	436.00	597.00
At 7 mm from center in superior	551.30 ± 36.14	545.80–556.81	553.00	455.00	642.00
At 7 mm from center in inferior	534.21 ± 35.80	528.76–539.67	534.00	450.00	626.00
At 7 mm from center in nasal	540.97 ± 35.33	535.59–546.35	541.50	451.00	634.00
At 7 mm from center in temporal	528.43 ± 34.22	523.22–533.64	529.00	438.00	607.00

SD = standard deviation.

The average IOP was 0.6, 0.47, 0.91, and 0.44 mm Hg lower than the primary position in upgaze, downgaze, medial gaze and lateral gaze (15°), respectively (*P* < .001). Similarly, IOP measured by the iCare IC200 at various locations on the cornea, 2 mm from the limbus in all 4 quadrants, was lower than IOP measured at the central cornea IOPs at 2 mm from the limbus in the inferior, nasal and temporal cornea at 0.5, 0.69 and 0.57 mm Hg lower than IOP measured at central cornea (*P* < .001). At the superior cornea, IOP did not significantly differ from IOP at the center (*P* = .83) (Table 2).

**Figure 5.**
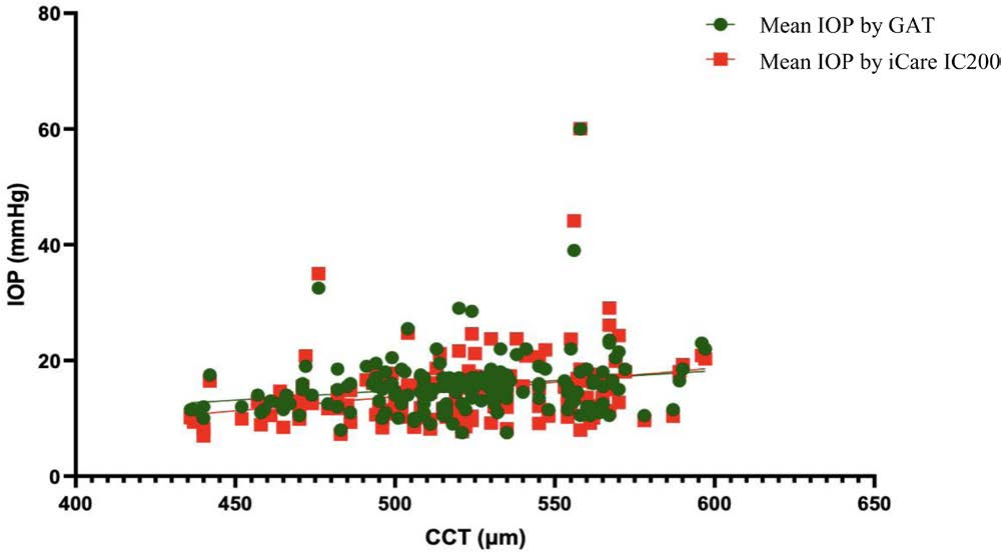
Scatter diagram shows the correlation between the CCT and IOP values measured using GAT and iCare IC200. CCT = central corneal thickness, IOP = intraocular pressure, GAT = Goldmann applanation tonometer.

A scatter diagram shows a good correlation between central corneal thickness and IOP values measured with GAT and iCare (Fig. 5). Linear regression analysis revealed a positive relationship between the central corneal thickness and IOP values among healthy patients, showing that the IOP measured by iCare at the central cornea will increase by 0.24 mm Hg for every 10 µm increase in CCT.

ICC of agreement between GAT and iCare IC200 was 0.96. Intra-observer measurements in different positions of the eye and different locations on cornea, ICC were all 0.99 (*P* < .001).

## 4. Discussion

A difference was observed between measurements in GAT and iCare IC200 for which IOP measured by GAT was higher than IOP measured by iCare at about 0.7 mm Hg and the standardized mean difference was 0.13 (−0.09 to 0.34) which is in the trivial range. The AUC values from our study was 0.98 which showed that the iCare IC200 accuracy was excellent. Other published data comparing the iCare rebound tonometer and GAT measurement indicated higher IOP values,^[4,6,7]^ lower IOP values^[8,9]^ and no difference between the 2 methods.^[2,10,11]^

The average IOP measurement using the iCare IC200 in various eye positions, namely, upgaze, downgaze, medial gaze and lateral gaze, was significantly lower than the IOP measured by the iCare IC200 at the primary position. The mean difference IOP was −0.6 mm Hg in upgaze, −0.47 mm Hg in downgaze, −0.91 mm Hg in medial gaze and −0.44 mm Hg in lateral gaze (*P* < .001), which constitute 15° angle deviated from the primary position. This result differed from a related study, reporting that IOP increased significantly in all-eye gaze positions compared with the primary position.^[11]^ In that study, IOP was measured during maximum eye gaze. We hypothesized that the disagreement was due to the difference in degrees of eye deviation. We examined IOP at a 15-degree deviated angle because we thought it comprised a common position error that could occur in real-world practice. Therefore, we can certainly about IOP results when the patient was not in primary position or had some degree of eye deviation. The difference was slightly lower that might be clinically not significance.

Several studies^[11–13]^ have compared the IOP using a rebound tonometer at the center and periphery of the cornea among humans. They measured the IOP using the rebound tonometer at the center and at 2 mm from the limbus in the nasal and temporal regions, with the probe perpendicular to the corneal plane. Gonzalez et al^[12]^ showed that nasal and temporal IOPs were 0.6 and 0.3 mm Hg lower than the central IOP, respectively (*P* < .05). Queiros et al^[13]^ demonstrated that nasal and temporal IOPs were 0.75 and 0.37 mm Hg lower than the central IOP (*P* < .05 and *P* > .05, respectively). Upon comparing our results, we noted that the nasal, temporal, and inferior IOPs were significantly lower than the center IOP with mean 0.69, 0.57, 0.5 mm Hg, respectively (*P* < .001). The IOP in the superior region at 2 mm from the limbus was lower but showed no statistically significant difference from the center IOP with a mean of 0.03 mm Hg (*P* = .83). Many biomechanical factors in the eye could explain this difference. For instance, histologically, the density of collagen fibrils in the peripheral cornea appears to be less compact than that in the central region, making it susceptible to corneal tensility and elasticity when measuring IOP.^[14]^ We postulated that it led to lower IOP readings in the peripheral cornea. Thus, the patient who had pathology at central cornea that impeded the measurement from GAT or iCare at the center, we can be reliable on measurement at the peripheral cornea. Though, the difference was slightly lower, it might not be significant in clinical practice.

Our study exhibited good intra-observer reliability for every measurement. However, this study encountered limitations. First, we measured IOP only at a 15-degree deviated angle from the primary position, which was a minimally deviated angle representing a common measurement error. Second, we measured IOP in the same order, which could have resulted in systematic error. However, the sequential nature of IOP measurements may not have affected the IOP results in this study because we paused a while between measurements using GAT and iCare and the physician measuring iCare was blinded to GAT readings. Third, in cases of ocular pathology, for example, muscle restriction, the IOP may not be compatible with our results. Further study may need to be conducted in special populations, for example among patients with thyroid eye disease and strabismus.

To our knowledge, corneal thickness influences the measurement of IOP. The association between IOP readings using the iCare IC200 and CCT among healthy patients illustrated that IOP would increase by 0.24 mm Hg for 10 µm increment in CCT. Min Chen^[6]^ The reported measurement error of 0.5 mm Hg with iCare pro per 10 µm increased in CCT among normal IOP patients. No related study analyzed the IOP measured using the iCare IC200 correlated with CCT.

## 5. Conclusion

The iCare IC200 is fully portable, easy to use, requires no anesthesia, and its freedom of positioning allows measurement in vary positions. When measurement in 15-degree deviated angles of the eye and 2 mm from limbus at the peripheral cornea, the difference of IOP were all in trivial range compare to IOP measured from the center which might be acceptable in clinical practice. Between iCare and GAT, the difference of IOP measurement was insignificant.

## Author contributions

Study conception and design: Wallop Iemsomboon, Raveewan Choontanom, Isaraporn Treesit.

Data collection: Sirada Wongwanwatana, Isaraporn Treesit.

Analysis and interpretation of results: Sirada Wongwanwatana, Isaraporn Treesit, Panrapee Funarunart.

Draft manuscript preparation: Sirada Wongwanwatana, Isaraporn Treesit, Panrapee Funarunart.

All authors reviewed the results and approved the final version of the manuscript.
